# The expanding burden of idiopathic intracranial hypertension

**DOI:** 10.1038/s41433-018-0238-5

**Published:** 2018-10-24

**Authors:** Susan P. Mollan, Magda Aguiar, Felicity Evison, Emma Frew, Alexandra J. Sinclair

**Affiliations:** 1 Birmingham Neuro-Ophthalmology Unit, University Hospitals Birmingham NHS Foundation Trust, Birmingham, B15 2TH UK; 2 Metabolic Neurology, Institute of Metabolism and Systems Research, University of Birmingham, Birmingham, B15 2TT UK; 3 Health Economics Unit, Institute of Applied Health Research, University of Birmingham, Birmingham, B12 2TT UK; 4 Department of Informatics, University Hospitals Birmingham NHS Trust, Queen Elizabeth Hospital Birmingham, Mindelsohn Way, Edgbaston, Birmingham, B15 2WB UK; 5 Centre for Endocrinology, Diabetes and Metabolism, Birmingham Health Partners, Birmingham, B15 2TH UK; 6 Department of Neurology, University Hospitals Birmingham NHS Foundation Trust, Birmingham, B15 2TH UK

## Abstract

**Objective:**

To quantify the hospital burden and health economic impact of idiopathic intracranial hypertension.

**Methods:**

Hospital Episode Statistics (HES) national data was extracted between 1st January 2002 and 31st December 2016. All those within England with a diagnosis of idiopathic intracranial hypertension were included. Those with secondary causes of raised intracranial pressure such as tumours, hydrocephalus and cerebral venous sinus thrombosis were excluded.

**Results:**

A total of 23,182 new IIH cases were diagnosed. Fifty-two percent resided in the most socially deprived areas (quintiles 1 and 2). Incidence rose between 2002 and 2016 from 2.3 to 4.7 per 100,000 in the general population. Peak incidence occurred in females aged 25 (15.2 per 100,000). 91.6% were treated medically, 7.6% had a cerebrospinal fluid diversion procedure, 0.7% underwent bariatric surgery and 0.1% had optic nerve sheath fenestration. Elective caesarean sections rates were significantly higher in IIH (16%) compared to the general population (9%), *p* < 0.005. Admission rates rose by 442% between 2002 and 2014, with 38% having repeated admissions in the year following diagnosis. Duration of hospital admission was 2.7 days (8.8 days for those having CSF diversion procedures). Costs rose from £9.2 to £50 million per annum over the study period with costs forecasts of £462 million per annum by 2030.

**Conclusions:**

IIH incidence is rising (by greater than 100% over the study), highest in areas of social deprivation and mirroring obesity trends. Re-admissions rates are high and growing yearly. The escalating population and financial burden of IIH has wide reaching implications for the health care system.

## Introduction

In light of the growing obesity epidemic, re-evaluation of trends in idiopathic intracranial hypertension (IIH) are needed. This would inform the agenda to standarise care pathways, improve quality of care provision and drive developments in novel therapeutic options to reduce the burden of this expanding disease.

IIH is a condition of unknown aetiology which occurs predominantly in obese women [1, 2]. There are currently few treatment options for IIH [3], management is typically medical, with those experiencing progressive visual loss undergoing surgical procedures [4]. Currently weight loss is the only disease modifiable therapy [5].

IIH was considered a rare condition. Previous annual incidence was reported at approximately 0.5–2 in 100,000 in the general population [6–12]. Prevalence data is sparse for the United Kingdom (UK) but a retrospective case review has reported prevalence of 10.9 per 100,000 for the general population in Sheffield, UK [6]. It has been widely speculated that the incidence of IIH is increasing in line with the world-wide epidemic of obesity [2, 10, 12].

Despite the relative rarity of IIH the multidisciplinary manifestations of the condition leads these patients to access hospital care though a large number of hospital specialties. In the UK suspected patients attend accident and emergency departments, are admitted to hospital or have procedures on ambulatory day care units. The scale of emergency room attendances, hospital admissions and day case care in the England has not been previously reported. Previous data from the United States has highlighted the economic burden of IIH [13, 14].

This observational study aimed to use Hospital Episode Statistics (HES) dataset to quantify incidence trends over time within England. We sought to define incidence by geographical distribution and socio-economic deprivation as well as determine the annual admission rates, management strategy (medical vs. surgical) as well as obstetric outcomes. The secondary aims were to conduct a health economic evaluation to establish the financial impact.

## Methods

### Study design and setting

This study was conducted through use of registered national data sets, and included all patients with IIH admitted for hospital care in England between 1st January 2002 and 31st December 2016. Data were obtained from the HES, an administrative dataset covering all NHS Trusts in England, which processes over 125 million admitted patient, outpatient and accident and emergency records each year; generating a log of each clinical episode taking place in NHS hospitals or NHS commissioned activity in the independent sector (private care). Admitted patient care episodes are defined as emergency room attendances, ambulatory care attendances (for example for lumbar puncture) and inpatient care [15].

Each record is anonymised and comprises specific demographic details of the admitted patient including age group, gender, ethnicity and geographical information such as where patients are treated and the area where they live (it is worth noting that body mass index data is not recorded). Data was checked for duplicate patient identifiers to ensure there was no double counting of entries. University Hospitals Birmingham NHS Foundation Trust holds a Data Re-Use Agreement for the interrogation of the HES. The research involved non-identifiable information, previously collected in the course of patient care and available for public use.

To access information pertaining to all IIH admissions, validated International Classification of Diseases, Tenth Revision, Clinical Modification (ICD-10-CM) codes and procedural classifications from the Office of Population, Censuses and Surveys Classification of Interventions and Procedures, 4th revision (OPCS-4) codes were used (supplementary file 1).

Exclusion criteria were applied to help refine the data and ensure against miscoding of secondary causes of raised intracranial pressure such as tumours, hydrocephalus and cerebral venous sinus thrombosis (supplementary file 1). Due to the very high number of admitted patient care and comorbidities, we exclude those with a history of dialysis, as the high admission rates would have potentially biased the results.

### Data collection

Patient demographics at the time of admitted patient care episodes were recorded and included gender; ethnicity; geographical regional location, as classed by the Government Office Region (GOR); and social deprivation indices based on the English index of multiple deprivation (IMD) 2010. The IMD is the official measure of relative deprivation (for neighbourhoods) in England and has been used frequently as a measure of relative deprivation to guide resource allocation and provision of services in the United Kingdom. Deprivation in this context refers to the relative disadvantage an individual experience living in a certain area. The IMD is based on 38 routinely collected indicators, aggregated into seven weighted domains to represent different dimensions of deprivation, namely income, employment, health and disability, education and skills, barriers to housing and services, crime and environment.

The IMD uses an area-based model at a low geography (average of 1500 people). Ranking the areas from 1 (most deprived area) to 32,844 (least deprived area) and quintiles are calculated dividing the ranking into five equal groups [16].

World Health Organisation (WHO) obesity data, only available up to 2014, was used to estimate UK obesity trends. Obesity is classed as a body mass index (BMI) ≥ 30 and is age-standardized in18 years+ by gender in the UK. National rates of obesity from Health survey for England 2015, were used for correlation of obesity rates per deprivation quintiles [17].

Visual complications and related surgical treatments, including cerebrospinal fluid diversion procedures, optic nerve sheath decompression and bariatric surgery, were recorded in this cohort. Cerebral venous stenting was initially scoped, but the multiple codes used to define one procedure varied greatly, and the data here would have been inconsistent and inaccurate. To investigate women’s health through pregnancy outcomes, the IIH cohort was matched against HES data from the general population for number of live births and mode of delivery.

### Economic model methods

To estimate the direct health care costs of IIH, a patient pathway model was developed based on the HES data presented here, and the Cambridge shunt registry [18]. Only direct health care resources associated with the diagnosis and treatment of IIH were included and no costs relating to the wider economy (such as days lost to work, childcare costs or travel to hospital) were calculated. The cost data applied to the health care resource use was taken from Optom [19], 2015–16 NHS reference costs [20] and the British National Formulary [21] (supplementary file 2).

The pathway model was developed to illustrate each step of the patient pathway for the first year and tree diagrams were constructed (supplementary files 3,4). In addition to those already cited, to predict future costs other sources were utilised [22, 23] (supplementary file 5).

### Statistical analysis

The data were initially explored through descriptive analysis of variables using *t* tests for quantitative variables and a *χ*^2^ test for categorical variables to compare different groups. Incidence and obesity data were normally distributed and pearson correlations were utilized. All statistical analyses were conducted using GraphPad Prism^TM^ (version 7.04).

## Results

**Fig. 1:**
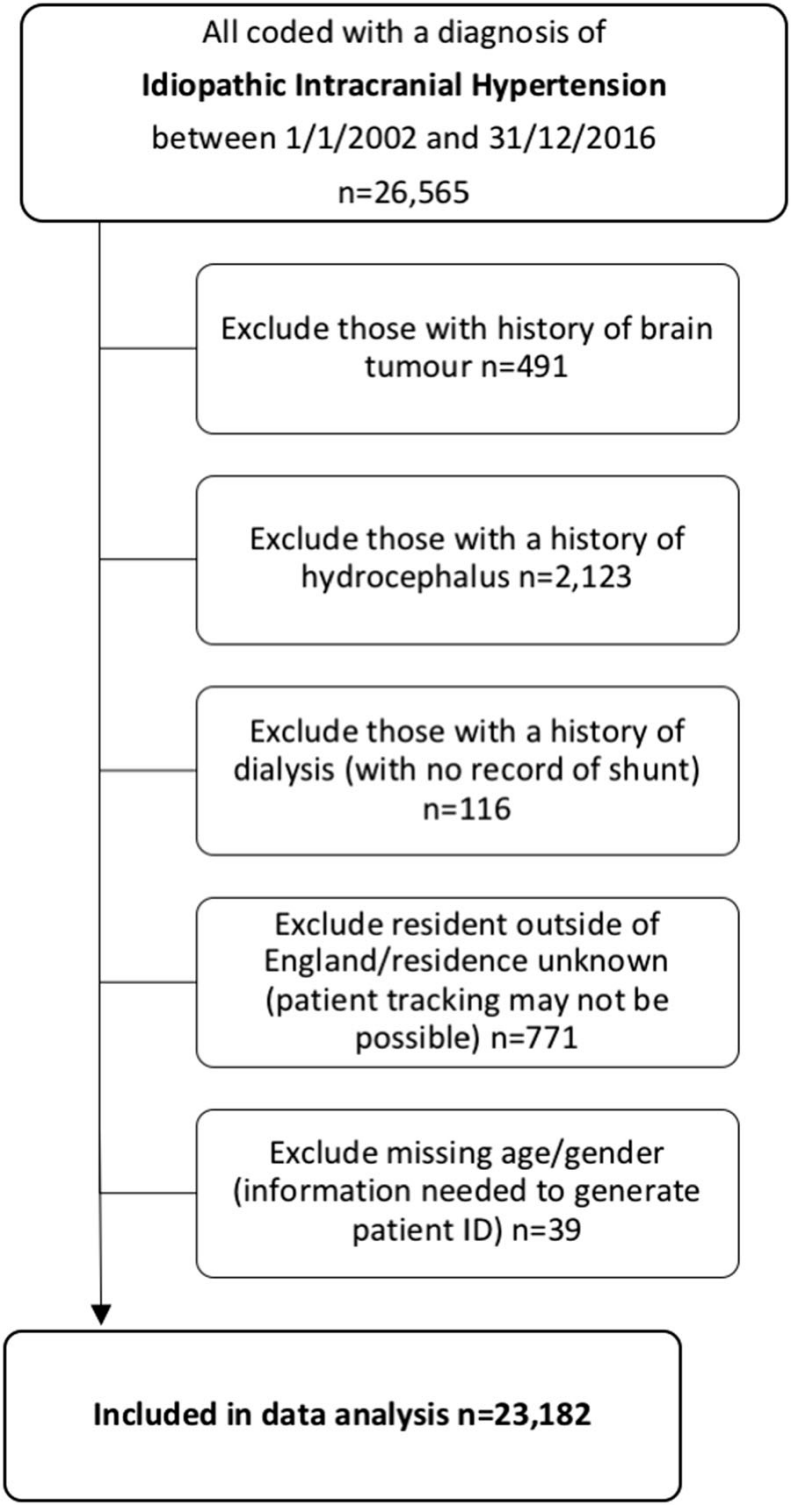
Consort diagram

HES identified 26,565 unique patients coded with IIH (ICD10 = G932) between 1/1/2002 and 31/12/2016. To ensure that an unbiased representative population was analysed 3383 were excluded from the analysis (Fig. 1).

**Fig. 2:**
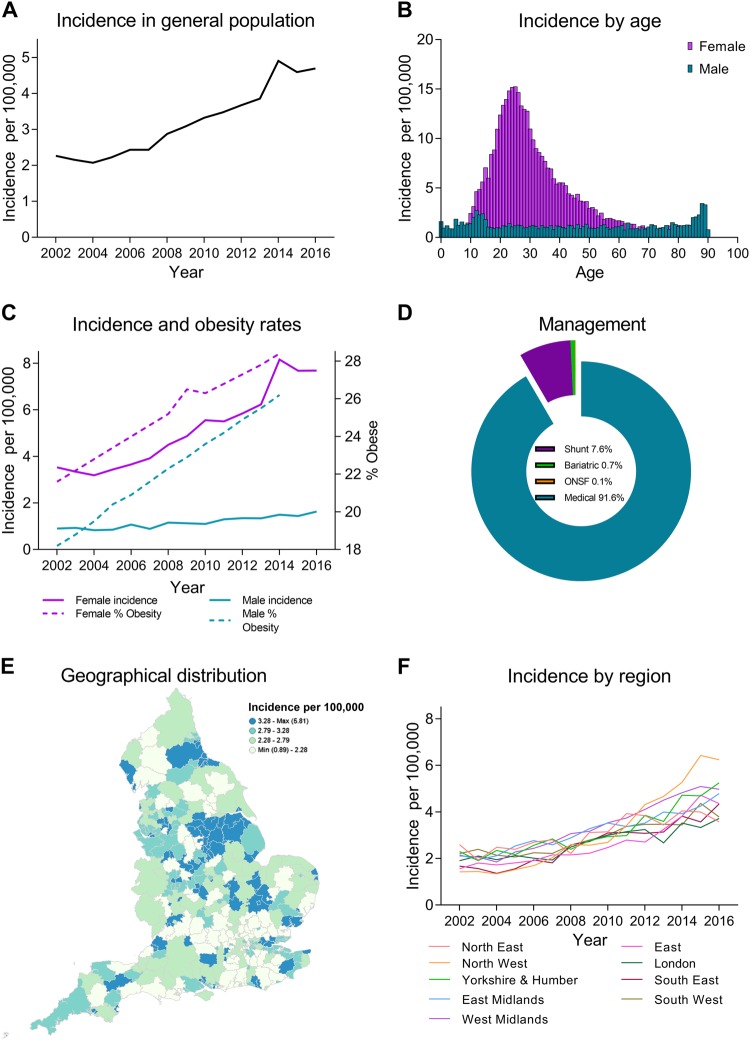
Composite figure. **a** Incidence in the general population. **b** Incidence by age and gender. **c** Annual incidence in females and males and Obesity rates (% obesity per annum (body mass index ≥ 30), age-standardized in 18 years + by gender in the United Kingdom. From World Health organisation http://apps.who.int/gho/data/node.main.A900A?lang=en Accessed 6 Oct 2017. **d** Management of IIH in the cohort. **e** Geographical distribution of diagnosed cases of IIH in England. **f** Distribution of cases by region per annum

The number of individual patients diagnosed with IIH was 23,182 during the study period (Fig. 2). 17.6% (4079/23,182) were male and 82.4% (19103/23,182) were female. The median age at diagnosis was 28 years (range: 21–40 years) (Fig. 2b; supplementary file 6).

With males having a higher median age of 32 years (range 14–50 years) and women a lower median age of 28 years (range: 22–38 years) (Fig. 2b). The majority of the ethnic groups reflected the population of England, with white being the prominent category in IIH (supplementary file 7).

### Incidence of IIH

In the general population, the incidence of IIH increased by 108% over the study period; in 2002 it was 2.26 per 100,000 rising to 4.69 per 100,000 in 2016 (Fig. 2a). Overall the peak incidence was seen in females aged 25 years and was 15.2 per 100,000 (Fig. 2b). The incidence in females in 2002 was 3.53 per 100,000 rising to 7.69 per 100,000 with the rates increasing in line with obesity in females (*r* = 0.914, *P* < 0.0001) and also in males (0.9 per 100,000 to 1.6 per 100,000, *r* = 0.913, *p* < 0.0001) (Fig. 2c). This represents a 118% increase in incidence in females.

**Fig. 3:**
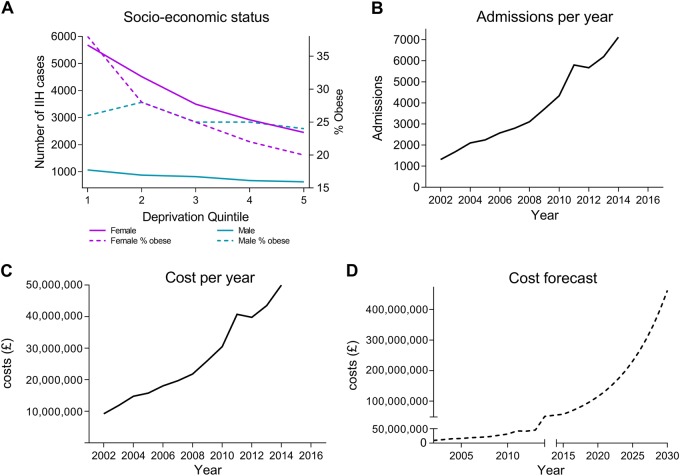
**a** IIH cases according to gender and socioeconomic status (solid line). % obesity rates (body mass index ≥ 30), age-standardised (aged > 16) by equivalised household income and sex (dotted line), data from Health Survey for England 2016. **b** Annual hospital episodes for IIH. **c** Hospital costs per annum for IIH. **d** Future predicted costs in millions (£) of IIH in England based on current trends

IIH incidence varied with geographical region across the UK with greater incidence noted in the East of England and the West Midlands (supplementary file 8, Figs. 2e, f). Cases of IIH varied with social deprivation (Fig. 3). Fewer cases are recorded in least deprived areas (deprivation quintile 5, 3080 (13.3%) cases of IIH). Whilst in areas of higher deprivation (deprivation quintiles 1 and 2) the majority of IIH cases were recorded (12,136 cases, 52.3%) (Fig. 3a).

The relationship between IIH and social deprivation varied with gender, with female cases significantly correlating with deprivation *r* = 0.89, *p* < 0.001, but this was not apparent in male cases (*r* = 0.37, *p* = 0.539) (Fig. 3a). BMI is known to differ with deprivation quintiles [17], and here amongst the female IIH population, cases correlated with deprivation quintile BMI (*r* = 0.98, *p* = 0.003), however male gender did not (*r* = 0.59, *p* = 0.291) (Fig. 3a).

### Admitted hospital episodes

Between 2002 and 2014 there were 47,982 hospital admitted patient care (emergency room attendances, admitted inpatient episodes and ambulatory care episodes) for IIH (Fig. 3b) for 23,182 patients. Over the study period there was an increase in admitted hospital episodes for IIH by 442% (1315 to 7123 patients admitted per year over the study period). Over half of the cohort had only one admission episode and no further admitted hospital care in the year following the diagnostic episode coded for IIH. 37.8% of the cohort had repeat hospital admissions in the first year following their diagnosis (supplementary file 9), with 0.9% having 10 or more admitted hospital episodes.

The average length of stay was 2.7 days, for those who had no surgical procedure, and for those who undergo neurosurgical management was 8.8 days. Over the study period, the majority were managed medically (91.6%); 7.64% (1771/23,182) had a shunt procedure; 0.68% (158/23,182) underwent bariatric surgery; and 0.07% (10/23,182) had an optic nerve sheath fenestration (Fig. 2d).

### Morbidity

Following diagnosis of IIH, according to HES records, 99 were coded as blind and 349 as visually impaired. Therefore, any type of visual impairment following a diagnosis of IIH is seen in 1.92% (445/23,182) of the cohort (with 0.42% being blind and 1.49% being visually impaired).

### Obstetric health

Those women between 16 and 55 years with IIH had similar birth rates to those in the general population of the same age range (supplementary file 10). IIH women were statistically less likely to have a normal delivery compared to age matched women in the general population with 4839 (57.44%) of women with IIH having a normal delivery compared to 3,612,199 (64.09%) in the general population, *p* < 0.005. Women with IIH were significantly more likely to undergo elective (*n* = 1328) and non-elective caesarean sections (*n* = 1427) compared to the general population (elective *n* = 514,287; non-elective *n* = 960,394), *p* < 0.005.

### Economic estimate

IIH was estimated to represent an annual hospital healthcare cost of £7016 per patient. When combined with the IIH population incidence rates, the total cost of IIH hospital care has risen dramatically from £9.2 million in 2002 to £49.9 million in 2014 (Fig. 3c). A prediction of future economic hospital burden was based on the current cost estimation and the expected population trends: if the rising trends of IIH continue then by 2030 it is projected to cost hospitals in England £462.7 million (Fig. 3d).

## Discussion

In this observational study of HES data the increase in IIH incidence by 108% is described. Of note the rising incidence significantly correlates with rising BMI in both genders, which has been previously speculated [4]. The incidence in women is four fold higher than in males (7.7 women vs. 1.6 per 100,000 male), with a peak incidence in women at the age of 25 years (15.2 per 100,000). Adult IIH has not been previously associated with social deprivation and adverse obstetric outcomes. These factors are new signals which reinforce that this disease should not be assumed “benign”.

Over half of the cohort was recorded in the lowest two quintiles of the IMD. Deprivation and social determinants are well known to cause a wide range of adverse health effects and are associated with higher morbidity and mortality [24, 25]. Area deprivation, as measured utilizing IMD, is an aggregated marker of social deprivation [25]. It is reliably used and has been associated with poorer clinical outcomes [26] and an increased number of co-morbidities [27, 28]. We have shown that IIH cases are significantly associated with deprivation quintile specific BMI rates (Fig. 3a) and we hypothesise that increased BMI may be a predominant factor in determining the variable in IIH social deprivation.

Over a third of the cohort had multiple hospital admissions (emergency room, inpatient or ambulatory care episodes) within the first year following diagnosis (Table 1); a 442% increase in admissions in the period studied. Deprivation status has been found to strongly influence admission and readmission rates for medical patients [29]. Primary care and unplanned hospital services are accessed more by those in deprived areas than those who are not [30] and those from deprived areas are less likely to visit a secondary care specialist [31]. National consensus guidelines could potentially help to reduce these attendances [32].

A prevalence study [33] reported 1–2% severe visual loss in IIH within the UK. The visual loss rates reported here are 1.92% of this English cohort, with 0.42% being blind and 1.49% with any kind of visual impairment. These rates may be an underestimation due to the differences in the definitions of visual impairment between the coding in HES and national definitions for certificate of visual impairment (supplementary file 1) [34]; and potentially represent underreporting of comorbidities in the HES data. Linkage of the HES and the certificate of visual impairment register was not possible with this anonymized study design.

The major strength of this study is that the analysis covers all IIH patients admitted hospital episodes in English NHS and private hospitals over a 14 year period and is therefore a unique population-wide assessment. Using national data over a long time-period allows for variation across the years. Unlike insurance company data our data is unlikely to be biased due to variation in funding between hospitals, insurance coverage or the patients’ ability to pay for care. However, like any database improvements in clinical record keeping and hence coding will improve the accuracy of the data. Likewise, we cannot be sure each of the individuals fulfilled the diagnostic criteria for IIH, and are dependant on the medical staff to make accurate diagnoses [1]. Changes in awareness of the condition and the medical literature, such as publication of revised diagnostic criteria [1], could have inflated the incidence following publication. Within the results there is a portion of patients diagnosed over the age of 65 years (supplementary Table 5), these cases likely represent misdiagnosis of phenotypical IIH, as the natural history of the disease is within the younger age ranges [2, 4].

Obstetric health is important in IIH, as the majority are women of child-bearing age (Fig. 2b). Literature in this area is case based [35]. Although those with IIH have similar parity compared to the general population (supplementary file 10) they were significantly more likely to undergo both elective and non-elective caesarean sections compared to the general population. This may be due to the association of obesity, as higher rates of caesarean section are reported in overweight and obese individuals [36]. Practical guidance in IIH does not suggest opting for caesarean section based solely on the diagnosis of IIH and as the transient elevations in ICP during the second stage of labour are unlikely to impact on the optic nerve function, except in the setting of fulminant IIH [32].

IIH is considered rare, however the rising incidence, hospital episodes and subsequent economic impact are noteworthy with wide reaching implications for health care service provision. The consensus guidelines [32] will start to shape and standardised care pathways and improve the quality of care. Development of novel therapeutic options may help to reduce the burden of this expanding disease.

### Summary

#### What was known before

- Previous annual incidence was reported at approximately 0.5–2 in 100,000 in the general population.
- It has been widely speculated that the incidence of IIH is increasing in line with the world-wide epidemic of obesity.
- No previous studies have determined IIH as a disease predominantly associated with social deprivation.

#### What this study adds

- In this study of 23,182 IIH patients incidence is rising (4.69 per 100,000 in 2016) and this in line with increasing body mass index rates.
- IIH is more commonly found in those from areas of social deprivation.
- The hospital economic burden has risen from 9.2 million in 2002 to 49.9 million in 2014.

## Electronic supplementary material

Table: Inclusions and exclusions codes used

Table of cost items used in cost estimation

Figure to show the typical patient pathway

Figure of the tree diagram to show the typical readmission pathway for patients

Methods used in the cost estimation

Table to show the age groups of the cohort

Table to show the ethnicity as recorded by HES

Table to show the regional distribution of cases and the social economic deprivation quintile

Table to show the number of admitted hospital episodes, following the admitted hospital episode where the diagnosis of IIH was made

Table showing the number of births in the IIH cohort compared to the general population in England as coded by HES
