# Supplementary material for: The expanding burden of idiopathic intracranial hypertension
Source: Eye (Lond). 2018 Oct 24;33(3):478–85. doi: 10.1038/s41433-018-0238-5 (PMC6460708; doi:10.1038/s41433-018-0238-5)
Supplement: Supplementary file 1 — Table: Inclusions and exclusions codes used [file 41433_2018_238_MOESM1_ESM.docx]

**Supplementary File 9:**

The number of admitted hospital episodes in the first year following a diagnosis of IIH.

| Number of additional admitted hospital attendances in first year following the initial attendance for the diagnosis of IIH | Number of patients (% of total number of patients) |
| --- | --- |
| 0 | 14504 (62.2) |
| 1 | 4105 (17.7) |
| 2 | 1916 (8.3) |
| 3 | 962 (4.1) |
| 4 | 519 (2.2) |
| 5 | 349 (1.5) |
| 6 | 261 (1.1) |
| 7 | 180 (0.8) |
| 8 | 92 (0.4) |
| 9 | 76 (0.3) |
| 10+ | 218 (0.9) |
