# Supplementary material for: The expanding burden of idiopathic intracranial hypertension
Source: Eye (Lond). 2018 Oct 24;33(3):478–85. doi: 10.1038/s41433-018-0238-5 (PMC6460708; doi:10.1038/s41433-018-0238-5)
Supplement: Supplementary file 2 — Table of cost items used in cost estimation [file 41433_2018_238_MOESM2_ESM.docx]

**Supplementary File 8:**

The regional location of the recorded home address, by Government Office Region (GOR), for each patient newly diagnosed with IIH (2002-2016) and their socio-economic deprivation quintile (based on Index of Multiple Deprivation 2010).

| **Region of residence (GOR)** | **Male**  **number (%)** | **Female number (%)** | **Total**  **number (%)** |
| --- | --- | --- | --- |
| East of England | 586 (14.4) | 2784 (14.6) | 3370 (14.5) |
| West Midlands | 646 (15.8) | 2686 (14.1) | 3332 (14.4) |
| Yorkshire and Humber | 627 (15.4) | 2582 (13.5) | 3209 (13.8) |
| South East | 551 (13.5) | 2254 (11.8) | 2805 (12.1) |
| South West | 365 (8.9) | 2126 (11.1) | 2491 (10.7) |
| North East | 366 (9.0) | 1931 (10.1) | 2297 (9.9) |
| London | 419 (10.3) | 1877 (9.8) | 2296 (9.9) |
| North West | 333 (8.2) | 1812 (9.5) | 2145 (9.3) |
| East Midlands | 179 (4.4) | 1044 (5.) | 1223 (5.3) |
| No Fixed Abode | 7 (0.2) | 7 (0.0) | 14 (0.1) |
| **Deprivation quintile** |  |  |  |
| 1 – most deprived | 1065 (26.1) | 5682 (29.7) | 6747 (29.1) |
| 2 | 874 (21.4) | 4515 (23.6) | 5389 (23.2) |
| 3 | 816 (20.0) | 3498 (18.3) | 4314 (18.6) |
| 4 | 675 (16.5) | 2918 (15.3) | 3593 (15.5) |
| 5 - Least deprived | 627 (15.4) | 2453 (12.8) | 3080 (13.3) |
| Unknown | 22 (0.6) | 37 (0.6) | 59 (0.3) |
