# Supplementary material for: The expanding burden of idiopathic intracranial hypertension
Source: Eye (Lond). 2018 Oct 24;33(3):478–85. doi: 10.1038/s41433-018-0238-5 (PMC6460708; doi:10.1038/s41433-018-0238-5)
Supplement: Supplementary file 3 — Figure to show the typical patient pathway [file 41433_2018_238_MOESM3_ESM.docx]

**Supplementary File 7:**

The ethnicity recorded by HES data of the IIH cohort (2002-2016), with a comparison made to the percentage ethnic groups in England and Wales in 2011.

| **Ethnicity** | **Males** | **Females** | **Persons** | **Percentage of total population** |
| --- | --- | --- | --- | --- |
| White | 2904 (71.2%) | 14419 (75.5%) | 17323 (74.7%) | 86.0% |
| Black/Black British | 125 (3.1%) | 751 (3.9%) | 876 (3.8%) | 3.3% |
| Asian/Asian British | 197 (4.8%) | 696 (3.6%) | 893 (3.9%) | 7.5% |
| Chinese | 7 (0.2%) | 20 (0.1%) | 27 (0.1%) | 0.7% |
| Mixed | 48 (1.2%) | 210 (1.1%) | 258 (1.1%) | 2.2% |
| Other | 78 (1.9%) | 267 (1.4%) | 345 (1.5%) | 1.0% |
| Unknown | 720 (17.7%) | 2740 (14.3%) | 3460 (14.9%) | - |
