# Supplementary material for: The expanding burden of idiopathic intracranial hypertension
Source: Eye (Lond). 2018 Oct 24;33(3):478–85. doi: 10.1038/s41433-018-0238-5 (PMC6460708; doi:10.1038/s41433-018-0238-5)
Supplement: Supplementary file 4 — Figure of the tree diagram to show the typical readmission pathway for patients [file 41433_2018_238_MOESM4_ESM.docx]

**Supplementary File 6:**

The age group of the IIH cohort (2002-2016).

|  | **Age (years)** | **Males** | **Females** | **Persons** |
| --- | --- | --- | --- | --- |
|  |  | Number (%) | | |
|  | Under 13 | 855 (21.0%) | 878 (4.6%) | 1733 (7.5%) |
|  | 13-16 | 361 (8.9%) | 1083 (5.7%) | 1444 (6.2%) |
|  | 17-19 | 133 (3.3%) | 1347 (7.1%) | 1480 (6.4%) |
|  | 20-24 | 289 (7.1%) | 3538 (18.5%) | 3827 (16.5%) |
|  | 25-29 | 267 (6.5%) | 3565 (18.7%) | 3832 (16.5%) |
|  | 30-34 | 282 (6.9%) | 2468 (12.9%) | 2750 (11.9%) |
|  | 35-44 | 563 (13.8%) | 3233 (16.9%) | 3796 (16.4%) |
|  | 45-54 | 516 (12.7%) | 1730 (9.1%) | 2246 (9.7%) |
|  | 55-64 | 390 (9.6%) | 726 (3.8%) | 1116 (4.8%) |
|  | 65+ | 423 (10.4%) | 535 (2.8%) | 958 (4.1%) |
|  | **Total** | 4079 | 19103 | 23182 |
