# Supplementary material for: The expanding burden of idiopathic intracranial hypertension
Source: Eye (Lond). 2018 Oct 24;33(3):478–85. doi: 10.1038/s41433-018-0238-5 (PMC6460708; doi:10.1038/s41433-018-0238-5)
Supplement: Supplementary file 5 — Methods used in the cost estimation [file 41433_2018_238_MOESM5_ESM.docx]

**Supplementary file 5**

**Methods for the prediction health care costs in IIH.**

For the first admission, the pathway assumed that all patients were referred to the accident and emergency department (Emergency room) via an optometry or optician practice who suspected papilledema. In the UK, the majority of these patients would be admitted to hospital, with a total length of stay being 2.7 days, as derived from the HES data (see results). Initial investigations at admission include one lumbar puncture, one brain imaging scan and venography (assumed to be 50% CT and 50% MRI). The majority of patients were discharged on acetazolamide, at a dosage of 1g per day, and 40% of these patients were assumed to discontinue drug therapy within a year due to adverse events^22^. Of the initial cohort of patients that were referred to the Emergency room, 6.6% had CSF diversion or shunting surgery and an average length of stay of 8.8 days (as derived from the HES data). Between 2002 and 2014, The Cambridge Shunt registry^18^ reported 3000 shunt procedures, for all conditions including IIH, each year with 1400 procedures in adults, of which 53% were primary and 47% revisions; the placement location was recorded at 76.51% being Ventricular-; 7.47% Lumbar- and 1.39% other.  The model therefore assumed a ratio of 10:1 ventricular:lumbar placement of the shunt device for IIH.

Costs associated with the first year follow up visits were estimated by assuming the following schedule: an ophthalmology appointment every 3 months (which is at fixed cost no matter what investigations are included); neurology appointment every four months and for patients who have undergone a neurosurgical procedure, one additional post-operative neurosurgery appointment.

The second pathway represents the likely care received for patients who were readmitted to hospital within the year following their diagnosis (supplementary file 4). This pathway assumes that readmitted patients who had previously undergone CSF diversion surgery have a higher probability (0.51) of requiring CSF revision surgery.^23^ If no previous CSF diversion/shunt surgery was undertaken, the probability of having a CSF diversion surgery is 1% (derived from HES results). The pathway then assumed the same follow-up visit schedule as before with one additional neurosurgery follow-up visit for those patients who had surgery on readmission.
