# Supplementary figures and images for: The expanding burden of idiopathic intracranial hypertension
Source: Eye (Lond). 2018 Oct 24;33(3):478–85. doi: 10.1038/s41433-018-0238-5 (PMC6460708; doi:10.1038/s41433-018-0238-5)

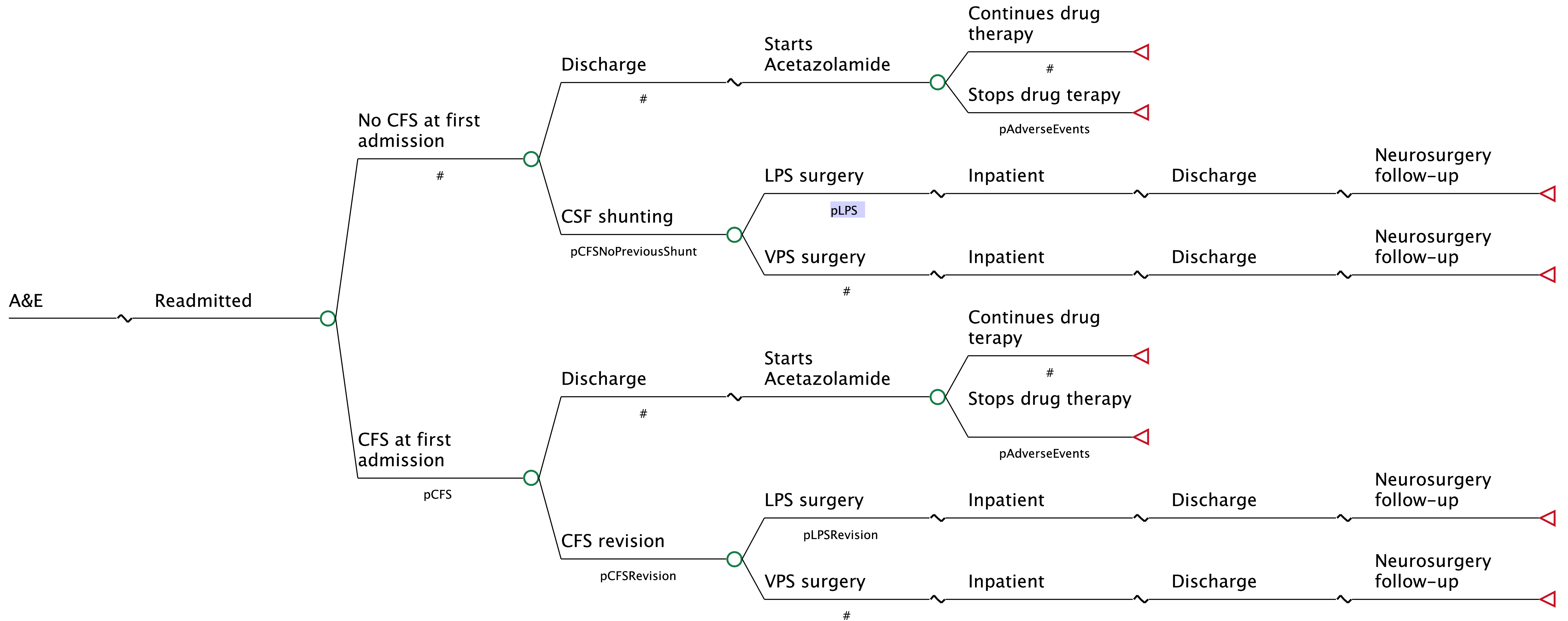

Supplement: Supplementary file 6 — Table to show the age groups of the cohort [file 41433_2018_238_MOESM6_ESM.pdf]

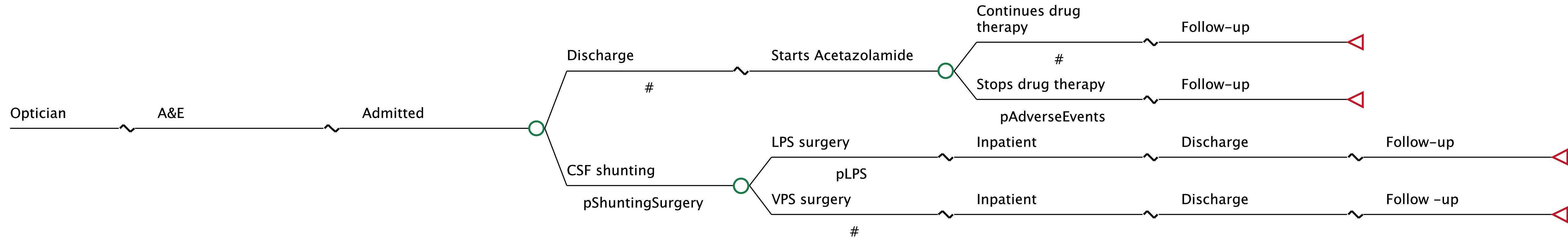

Supplement: Supplementary file 7 — Table to show the ethnicity as recorded by HES [file 41433_2018_238_MOESM7_ESM.pdf]
