# Supplementary material for: The expanding burden of idiopathic intracranial hypertension
Source: Eye (Lond). 2018 Oct 24;33(3):478–85. doi: 10.1038/s41433-018-0238-5 (PMC6460708; doi:10.1038/s41433-018-0238-5)
Supplement: Supplementary file 8 — Table to show the regional distribution of cases and the social economic deprivation quintile [file 41433_2018_238_MOESM8_ESM.docx]

**Supplementary file 2:**

Summary table of all costs included in the economic model analysis and source material from which the individual costs are derived. The model assumes same cost for first shunting surgeries and revision surgeries. Analysis includes direct costs only, related to secondary care and drug therapy prescribed in hospital.

| **Variable** | **Cost (£)** | **Code** | **Source** |
| --- | --- | --- | --- |
| **Optician visit** | 20.00 | NA | UK Health Centre. 2016 [1] |
| **Accident and Emergency attendance** | 138.00 | NA | NHS Reference Costs 2015-2016 [2] |
| **CT Head Scan** | 111.00 | RD24Z | NHS Reference Costs 2015-2016 [2] |
| **MRI Scan (head and orbit with intravenous contrast)** | 166.00 | RD05Z | NHS Reference Costs 2015-2016 [2] |
| **Lumbar puncture** | 664.01 | AA55 | NHS Reference Costs 2015-2016 [2] |
| **Venography** | 154.50* | U117 and U119 | NHS Reference Costs 2015-2016 [2] |
| **Ophthalmology visit (led by consultant)** | 94.18 | 130 | NHS Reference Costs 2015-2016 [2] |
| **Neurology (led by consultant)** | 178.94 | 400 | NHS Reference Costs 2015-2016 [2] |
| **Neurosurgery(led by consultant)** | 204.31 | 150 | NHS Reference Costs 2015-2016 [2] |
| **Optical Coherence tomography (OCT) scan** | 52.94 | RD40Z | NHS Reference Costs 2015-2016 [2] |
| **Humphrey visual field** | Included in Ophthalmology outpatient cost | NA | NHS Reference Costs 2015-2016 [2] |
| **Excess bed day** | 306.00 | NA | NHS Reference Costs 2015-2016 [2] |
| **Acetazolamide (cost per year)** | 663.20 | NA | British National Formulary 2016 [3] |
| **Lumbar Peritoneal Shunt (LPS)** | 4344.99 | HC71Z | NHS Reference Costs 2015-2016 [2] |
| **Ventricular Peritoneal Shunt (VPS)** | 8770.00 | AA52 | NHS Reference Costs 2015-2016 [2] |

**NB** All costs are indexed for GBP 2016 prices. * 50% were MRV and 50% CTVs.

1. UK Health Centre. 2016 (<http://www.healthcentre.org.uk/opticians/opticians-opticians-costs.html)> Last accessed 19^th^ December, 2017.
2. Department of Health. NHS reference costs 2015 to 2016: National schedule of reference costs. 2016. Last accessed 19^th^ December, 2017.
3. British National Formulary 2016 (<https://bnf.nice.org.uk/medicinal-forms/acetazolamide.html)>. Last accessed 19^th^ December, 2017.
