# Supplementary material for: The expanding burden of idiopathic intracranial hypertension
Source: Eye (Lond). 2018 Oct 24;33(3):478–85. doi: 10.1038/s41433-018-0238-5 (PMC6460708; doi:10.1038/s41433-018-0238-5)
Supplement: Supplementary file 9 — Table to show the number of admitted hospital episodes, following the admitted hospital episode where the diagnosis of IIH was made [file 41433_2018_238_MOESM9_ESM.docx]

**Supplementary File 1:**

Inclusion and exclusion codes used in the search strategy. To access information pertaining to all IIH admissions, validated International Classification of Diseases, Tenth Revision, Clinical Modification (ICD-10-CM) codes and procedural classifications from the Office of Population Censuses and Surveys Classification of Interventions and Procedures, 4th revision (OPCS-4) codes were used.

| Variable | Inclusion Codes | Exclusion Codes |
| --- | --- | --- |
| Idiopathic Intracranial Hypertension | ICD-10-CM code:  Benign Intracranial hypertension –G93.2  [Hypertension, idiopathic intracranial; Increased intracranial pressure; Pseudotumor cerebri; Raised intracranial pressure] | ICD-10-CM codes:  Hydrocephalus G91;  Cerebral venous sinus thrombosis G08;  Brain Cancer C70, C71;  Hypertensive  encephalopathy I67.4 |
| Visual outcomes | ICD-10-CM codes:  H540 - Blindness, binocular  H541 - Severe visual impairment, binocular  H542 - Moderate visual impairment, binocular  H544 - Blindness, monocular  H545 - Severe visual impairment, monocular  H546 - Moderate visual impairment, monocular  H547 - Unspecified visual loss.  H549 - Unspecified visual impairment (binocular) | ICD-10-CM codes:  H53.9 visual disturbance, unspecified  H470 - Disorders of optic nerve, not elsewhere classified  H543 - Mild or no visual impairment, binocular |
| Surgical history- Bariatric Surgery | OPCS-4 codes:  G301 G30.1 Gastroplasty NEC  G302 G30.2 Partitioning of stomach NEC  G303 G30.3 Partitioning of stomach using band  G304 G30.4 Partitioning of stomach using staples |  |
| Surgical history- Cerebrospinal fluid diversion procedures | OPCS-4 codes:  A122 A12.2 Creation of ventriculovascular shunt  A123 A12.3 Creation of ventriculopleural shunt  A124 A12.4 Creation of ventriculoperitoneal shunt  A53 A53 Drainage of spinal canal  A534 A53.4 Creation of lumboperitoneal shunt  A536 A53.6 Creation of lumbar subcutaneous shunt |  |
| Obstetric History | All deliveries- record of ICD-10-CM codes  O80-O84  OPCS-4 codes for all types of delivery of baby (R17-24) including:  Elective C-section R17  Other caesarean delivery R18 (in labour/emergency)  R21 Forceps Cephalic deliveries  R21 Forceps delivery  R22 Vacuum delivery  R24 Normal delivery  R24.9 All normal delivery | The data was restricted to only 1 birth in a 9 month period. |
