# Supplementary material for: The expanding burden of idiopathic intracranial hypertension
Source: Eye (Lond). 2018 Oct 24;33(3):478–85. doi: 10.1038/s41433-018-0238-5 (PMC6460708; doi:10.1038/s41433-018-0238-5)
Supplement: Supplementary file 10 — Table showing the number of births in the IIH cohort compared to the general population in England as coded by HES [file 41433_2018_238_MOESM10_ESM.docx]

**Supplementary file 10:**

Number of births in the general population (aged 16-55) compared to those diagnosed with IIH between 1^st^ January 2002 and 31^st^ December 2015.

| **Number of births** | **General population (excluding IIH** | **IIH** |
| --- | --- | --- |
| **1** | 2,927,954 (58.1%) | 2,535 (50.8%) |
| **2** | 1,633,387 (32.4%) | 1,714 (34.33%) |
| **3** | 376,778 (7.5%) | 569 (11.4%) |
| **4** | 77,495 (1.5%) | 134 (2.7%) |
| **5** | 15,841 (0.3%) | 28 (0.6%) |
| **6** | 3,598 (0.1%) | 11 (0.2%) |
| **7** | 865 (0.0%) |  |
| **8** | 197 (0.0%) |  |
| **9** | 46 (0.0%) |  |
| **10** | 7 (0.0%) |  |
